# Induction of macrophage pyroptosis-related factors by pathogenic *E. coli* high pathogenicity island (HPI) in Yunnan Saba pigs

**DOI:** 10.1186/s12917-021-02824-x

**Published:** 2021-03-07

**Authors:** Chunlan Shan, Shushu Miao, Chaoying Liu, Bo Zhang, Weiwei Zhao, Hao Wang, Wei Yang, Jinlong Cha, Ru Zhao, Peng Xiao, Hong Gao

**Affiliations:** 1 College of Animal Science and Technology, Yunnan Agricultural University, Kunming, 650201 China; 2 College of Veterinary Medicine, Yunnan Agricultural University, Kunming, 650201 China; 3 College of Food Science and Technology, Yunnan Agricultural University, Kunming, 650201 China

**Keywords:** Pathogenic *E. coli*, HPI, Macrophage, Pyroptosis, Caspase-1

## Abstract

**Background:**

Pyroptosis plays a pivotal role in the pathogenesis of many inflammatory diseases. The molecular mechanism by which pyroptosis is induced in macrophages following infection with pathogenic *E. coli* high pathogenicity island (HPI) will be evaluated in our study.

**Results:**

After infection with the HPI^+^/HPI^−^ strains and LPS, decreased macrophage cell membrane permeability and integrity were demonstrated with propidium iodide (PI) staining and the lactate dehydrogenase (LDH) assay. HPI^+^/HPI^−^-infection was accompanied by upregulated expression levels of NLRP3, ASC, caspase-1, IL-1β, IL-18 and GSDMD, with significantly higher levels detected in the HPI^+^ group compared to those in the HPI^−^ group (*P <* 0.01 or *P <* 0.05). HPI^+^ strain is more pathogenic than HPI^−^ strain.

**Conclusion:**

Our findings indicate that pathogenic *E. coli* HPI infection of Saba pigs causes pyroptosis of macrophages characterized by upregulated expression of pyroptosis key factors in the NLRP3/ASC/caspase-1 signaling pathway, direct cell membrane pore formation, and secretion of the inflammatory factor IL-1β and IL-18 downstream of NLRP3 and caspase-1 activation to enhance the inflammatory response.

## Background

*Escherichia coli* (*E. coli*), which is a typical Gram-negative member of the coliform genus *Escherichia*, plays a key role in the intestinal symbiosis of warm-blooded animals [1, 2]. Pathogenic *E. coli* strains cause serious harm to human and animal health, often causing severe diarrhea and septicemia [3]. High pathogenicity island (HPI) is a vital factor for the toxicity and pathogenicity of *E. coli*, and other highly pathogenic strains. It was first discovered in *Yersinia* [4] and is present only in virulent strains [5]. HPI has a functional core region, containing the *irp2*-*irp1*-*irp3*-*irp4*-*irp5*-*FyuA* gene axis known as the *irp2*-*FyuA* gene cluster, and the *irp2* marker gene [6]. Paauw et al. demonstrated that *Yersinia* containing HPI was more virulent by studying the iron carrier encoding HPI [7].

Pyroptosis is a form of programmed cell death (PCD) mediated by gasdermin D (GSDMD), relied on caspase-1, which induces cell swelling and is characterized by the rupture and release of cellular contents leading to an intense inflammatory reaction that is essential for the control of microbial infections [8, 9]. Several characteristics of pyroptosis appear to overlap with apoptosis, although the processes are distinct. Pyroptosis is similar to apoptosis [10] in that the small molecule dye propidium iodide (PI) enters the cell through the interstitial space in the plasma membrane and stains the nucleus [11]. Therefore, the formation of membrane pores in the plasma membrane was identified by PI staining. Pyroptosis is one of the host’s natural immune defense mechanisms against intracellular pathogen infection [12]. Activated caspase-1 and its precursors cleave GSDMD protein induce pyroptosis, and forms specific membrane pores with an inner diameter of 12–14 nm [13]. These pores disrupt the ion concentration gradient across the cell membrane and increase the permeability of the cell membrane, leading to swelling and disintegration of the cells [14–16].

NOD-like receptor family pyrin domain-containing 3 (NLRP3) inflammasomes are mainly composed of intracellular pattern-recognition receptor NLRP3, pre-caspase-1 and apoptosis-associated speck-like protein (ASC) [17], which can be activated by bacteria, viruses, fungi and apoptosis [18], and can also directly stimulate the caspase-1 domain activation. The activated caspase-1 then cleaves inactive pro-interleukin-1β (pro-IL-1β) and pro-interleukin-18 (pro-IL-18) to generate the active pro-inflammatory cytokines IL-1β and IL-18 [19], then released from the cytoplasm through this cell membrane pore [13]. Other immune cells are recruited and stimulated by IL-1β and IL-18, thereby inducing the synthesis of other inflammatory cytokines and enhancing the local and systemic inflammatory response [20].

It has been confirmed that *Shigella flexneri*, *Salmonella*, *Listeria*, *Pseudomonas aeruginosa*, *Francis tularensis*, *Legionella pneumophila* and *Yersinia* induce caspase-1-dependent pyroptosis in macrophages [20]. During infection, pyroptosis induces the death of host cells, which is an important process by which the growth and reproduction of the pathogenic microorganism is limited and the infection is cleared, thus providing effective protection of the host. Pyroptosis participates in the control of various bacterial infectious diseases. However, its mechanism and regulation mechanism has not been elucidated. Saba pigs are a breed local to Yunnan Province that are reared for their high rate of piglet production and an excellent meat quality. Saba sows are commonly used in hybrid breeding systems in central Yunnan Province [21].

To elucidate the pathogenic mechanism of *E. coli*, we evaluated the ability of pathogenic *E. coli* HPI to induce pyroptosis in macrophages, and investigated the underlying mechanism. Hu et al. demonstrated that LPS-activated cells, further stimulated by ATP, induced the activation of inflammatory factors and thereby induced pyroptosis [22]. Therefore, LPS + ATP was used as a positive reference to confirm the occurrence of pyroptosis in our study. In addition, we explored the effects of HPI^+^ and HPI^−^ strains on host cell infection. This information will provide an in-depth understanding of the mechanism of pyroptosis-related diseases, and highlight new therapeutic targets for clinical treatment of related diseases.

## Results

### Isolation and identification of pathogenic *E. coli* HPI

**Fig. 1:**
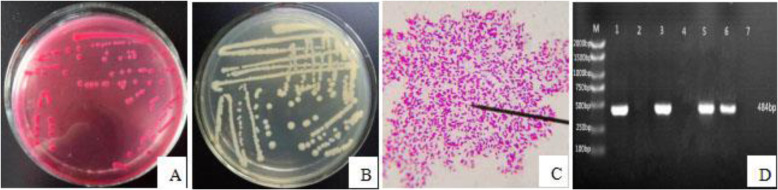
**a**: Isolation of pathogenic *E. coli*; **b:** Purification of pathogenic *E. coli*; **c:** Microscopic examination of pathogenic *E. coli* (1000′ magnification); **d**: The PCR amplification of HPI *irp2* gene (M: DL2000 Mark; 1–6: Experimental strains; 7: Negative control)

The *E. coli* HPI strains were cultured for 24 h until bright pink round colonies with smooth and moist surface and flat, neat edges were observed (Fig. 1a). Translucent raised colonies that were round in shape and with a smooth, moist surface were formed on normal nutrient agar medium (Fig. 1b). *E. coli* were identified as Gram-positive rod-shaped red cells (Fig. 1c). The presence of the *irp2* gene in the isolates was determined by PCR amplification using the extracted DNA as a template. In total, 43 of the 96 isolates were *irp2* gene positive (44.8%) and 53 were negative (55.2%). Representative results for PCR amplification of the HPI *irp2* gene are shown in Fig. 1d.

### MTT analysis of cell viability

**Fig. 2:**
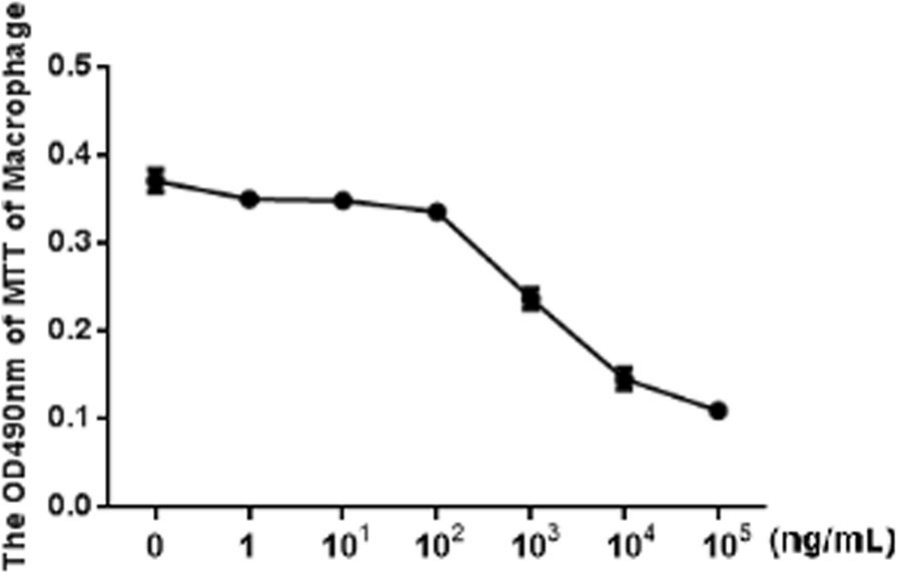
The determination of IC50 for LPS: the content of LPS (ng/mL)

The OD490 of macrophages at 24 h decreased as the concentration of LPS increased. The IC_50_ for LPS was calculated to be approximately 4.79 ng/mL according to the Improved Kou method (Fig. 2). This concentration of LPS was used in subsequent experiments.

### PI staining and concentrations of lactate dehydrogenase (LDH)

**Fig. 3:**
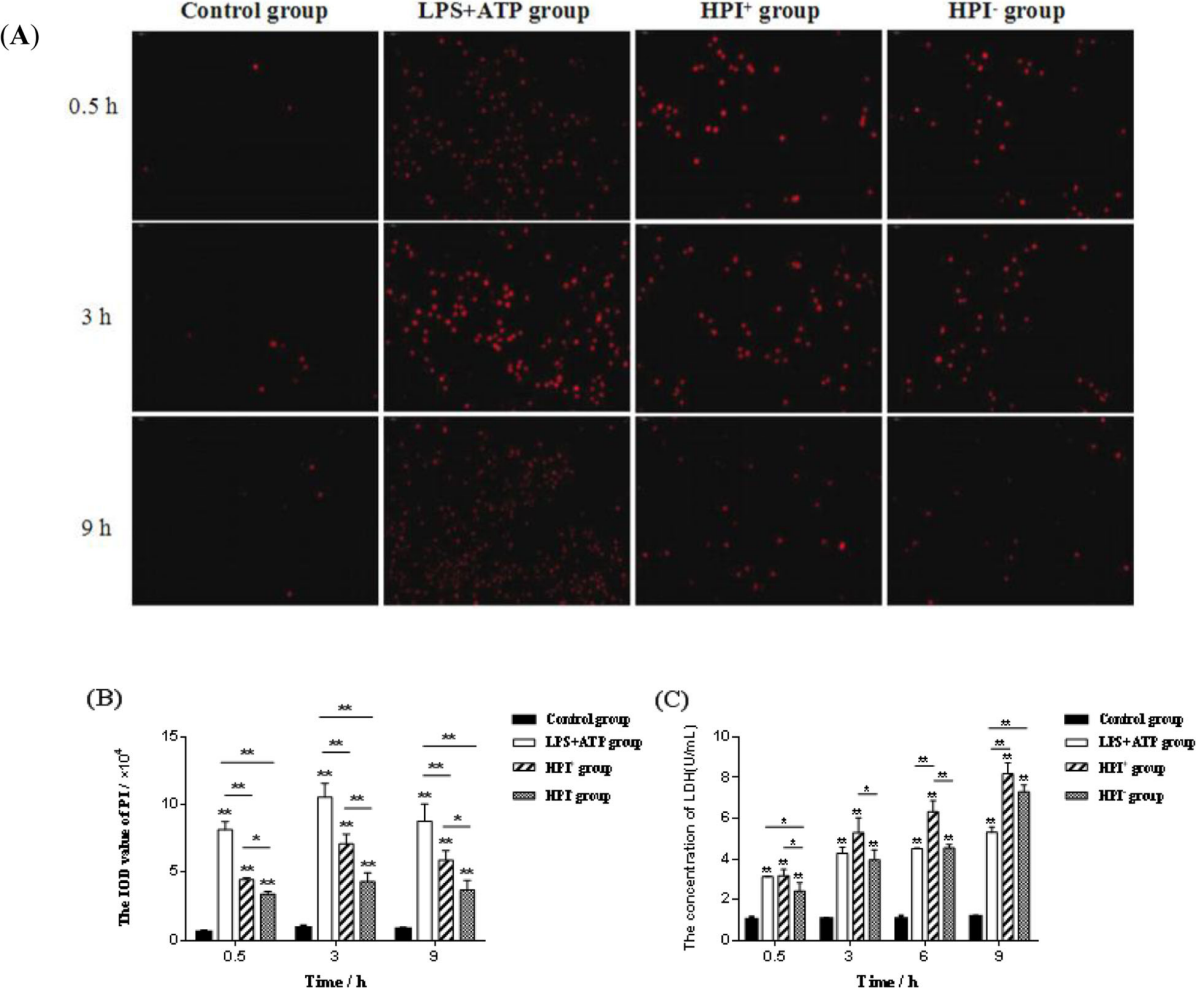
**a:** PI staining of macrophages after *E. coli* infection (200 × magnification, PI staining, red); **b:** The IOD value of PI in macrophages at 0.5 h, 3 h and 9 h after *E. coli* HPI infection. **c:** The concentration of LDH in macrophages at 0.5, 3, 6 and 9 h after *E. coli* HPI infection*.* *P < 0.05, **P < 0.01, *n* = 3

The results of PI staining are shown in Fig. 3a. The integrated optical density (IOD) values representing the intensity of PI staining of the HPI groups were significantly higher than that of the control group during the period of 0.5 to 9 h post-infection (*P <* 0.01). The IOD value of the LPS + ATP group was higher than those in the other groups after infection (*P <* 0.01). The IOD values in the HPI^+^ group were markedly higher than those in the HPI^−^ group at different time-points after *E. coli* infection, with significant differences detected at 0.5 and 9 h (*P <* 0.05) and extremely significant differences detected at 3 h (*P <* 0.01) (Fig. 3b). As shown in Fig. 3c, compared to the control group, the LDH concentrations in macrophages in the infection groups were significantly higher during the period of 0.5 to 9 h post-infection (*P* < 0.01). The LDH contents in the HPI^+^ group were markedly higher than those in the HPI^−^ group and extremely significantly higher than those detected at 6 h (*P* < 0.01). After macrophage infection, the IOD values of PI staining and LDH concentrations increased, indicating that HPI caused cell membrane rupture and changes in cell permeability.

### Effects of HPI infection on the mRNA expression of key pyroptosis genes in macrophages

**Fig. 4:**
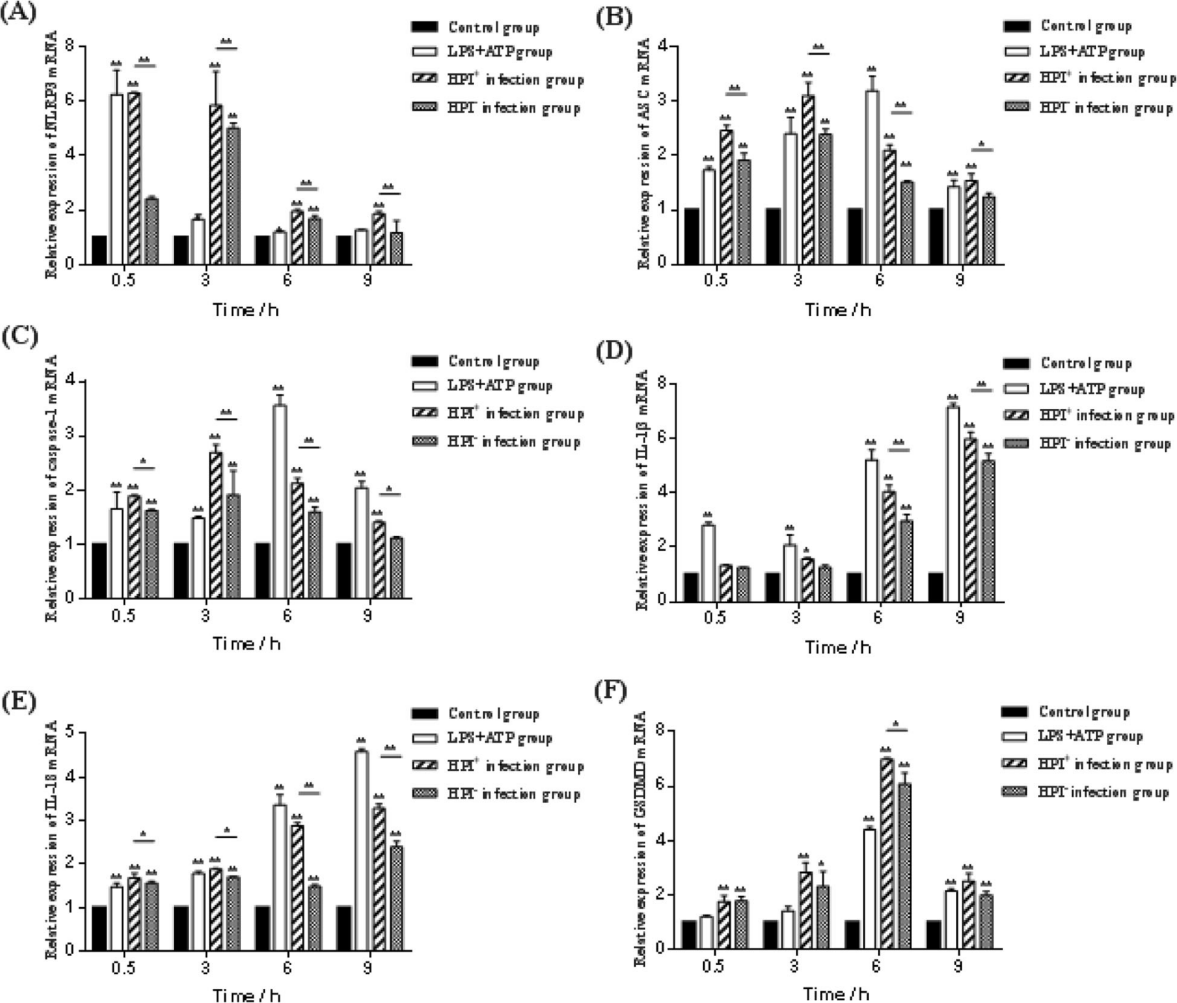
Relative mRNA expression of NLRP3 **a**, ASC **b**, caspase-1 **c**, IL-1β **d**, IL-18 **e** and GSDMD **f** in macrophages at 0.5 h, 3 h and 9 h after *E. coli* HPI infection. *P < 0.05, **P < 0.01, *n* = 3

The NLRP3 inflammasome is composed of NLRP3, ASC and pre-caspase-1. As shown in Fig. 4a, b and c, the relative expression of NLRP3, ASC and caspase-1 mRNA was generally upregulated first and then downregulated in the infection groups. NLRP3, ASC and caspase-1 mRNA expression in the HPI infection groups was significantly higher than that in the control at all time point post-infection (*P* < 0.01, *P* < 0.05 or *P* > 0.05). In addition, there were significant differences in the three genes mRNA expression between the HPI^+^ and the HPI^−^ groups after infection (*P* < 0.01 or *P* < 0.05). At 3 h post-infection, the relative expression of ASC and caspase-1 mRNA in the HPI infection groups was higher than that at the other time-points. As shown in Fig. 4d and e, the expression of IL-1β and IL-18 mRNA in the three infection groups was higher than that in the control group at 6 and 9 h post-infection (*P* < 0.01), with the highest expression at 9 h post-infection. The IL-1β and IL-18 expression levels in the HPI^+^ group were significantly different from those in the control and HPI^−^ groups at 6 and 9 h post-infection (*P* < 0.01). The increased expression levels of these five genes confirmed that HPI induced pyroptosis of macrophages. As shown in Fig. 4f, the expression of GSDMD mRNA in the infection groups was higher than that in the control group, significantly different at 6 and 9 h post-infection (*P* < 0.01). GSDMD mRNA levels in the HPI^+^ group were significantly higher than those in the HPI^−^ group at 6 h (*P* < 0.01). The highest expression of GSDMD at 6 h post-infection indicated that pyroptosis was the most severe at this time.

### Immunofluorescence assay (IFA) of NLRP3 and caspase-1 protein expression in macrophages

**Fig. 5:**
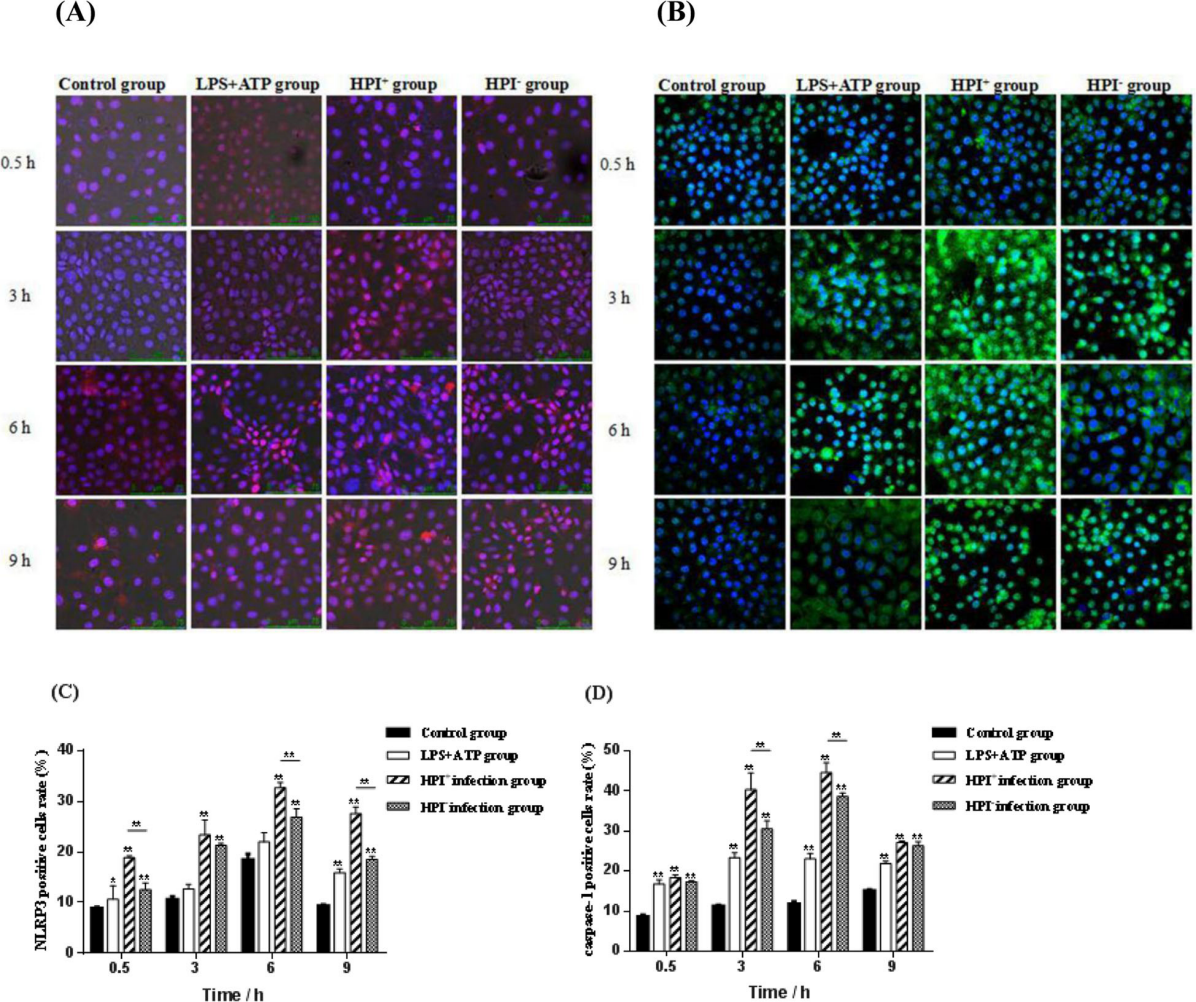
**a:** NLRP3 protein in macrophages at 0.5, 3, 6 and 9 h after *E. coli* HPI infection (NLRP3 staining, red; nuclear staining, blue); **b**: Caspase-1 protein in macrophages at 0.5, 3, 6 and 9 h after *E. coli* HPI infection (caspase-1 staining, green; nuclear staining, blue) **c**: Number of NLRP3 positive cells at 0.5, 3, 6 and 9 h after *E. coli* HPI infection. **d**: Number of caspase-1 positive cells at 0.5, 3, 6 and 9 h after *E. coli* HPI infection. *P < 0.05, **P < 0.01, *n* = 3

Immunofluorescence analysis of NLRP3 and caspase-1 expression in macrophages is shown in Fig. 5a and b. The positive cell rate of NLRP3 and caspase-1 in the three infection groups increased first and then decreased during the period from 0.5 to 9 h post-infection (Fig. 5c and d). At all time point post-infection, the positive cell rate of NLRP3 and caspase-1 in the HPI infection groups were significantly higher than those in the control group (*P <* 0.01). Furthermore, compared with the HPI^−^ group, the NLRP3 positive cell rate in the HPI^+^ group were significantly higher (*P <* 0.01) at 0.5, 6 and 9 h after infection, the caspase-1 positive cell rate in the HPI^+^ group were significantly higher (*P <* 0.01) at 3 and 6 h after infection.

### ELISA analysis of IL-1β and IL-18 levels in macrophages after *E. coli* infection

**Fig. 6:**
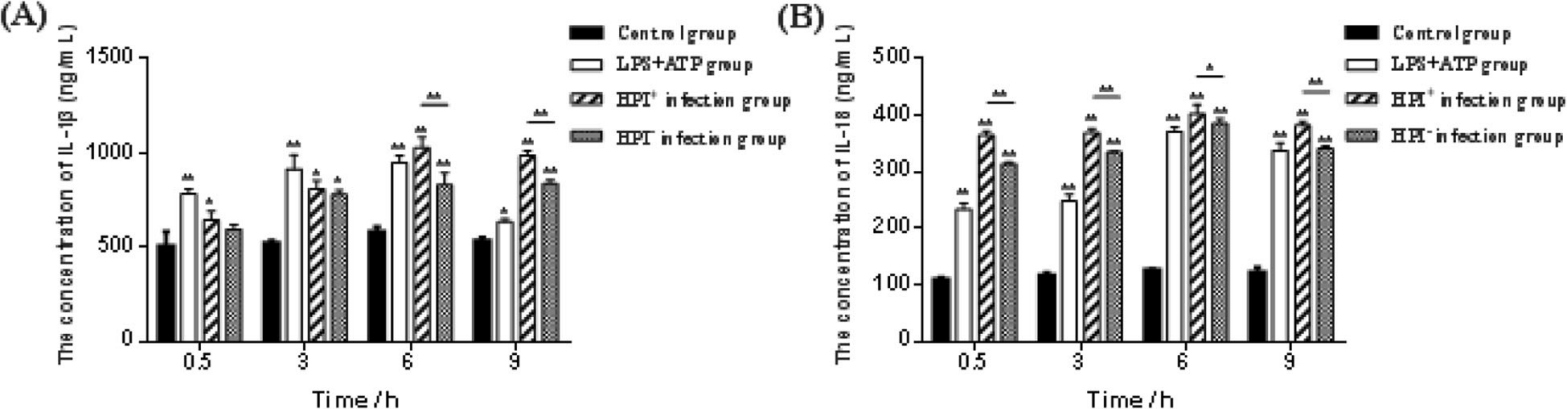
**a:**The concentration of IL-1β in macrophages at 0.5, 3, 6 and 9 h after *E. coli* HPI infection*.* **b***:* The concentration of IL-18 in macrophages at 0.5, 3, 6 and 9 h after *E. coli* HPI infection*.* *P < 0.05, **P < 0.01, *n* = 3

The concentrations of IL-1β and IL-18 in the culture supernatants of macrophages were detected by ELISA (Fig. 6a and b). The concentrations both cytokines were initially upregulated followed by a gradual decline in expression with time after infection. The IL-1β and IL-18 levels in the LPS + ATP group were significantly higher than those in the control group at 0.5, 3 and 6 h post-infection (*P* < 0.01). The IL-1β and IL-18 content in the control group was significantly lower than those in the HPI infection groups at 6 and 9 h post-infection (*P <* 0.01). The concentrations of IL-1β in the HPI^+^ group were significantly higher than those in the HPI^−^ group at 6 and 9 h post-infection (*P <* 0.01), the concentrations of IL-18 in the HPI^+^ group were significantly higher than those in the HPI^−^ group at 0.5, 3 and 9 h post-infection (*P <* 0.01) and higher than those in the HPI^−^ at the other time-points (*P <* 0.05).

## Discussion

Pyroptosis is a form of innate immune defense against intracellular bacteria [23]. In contrast to apoptosis, numerous pores (1–2 nm) are formed on the cell membrane of pyroptotic cells, leading to the release of the cellular contents and inflammatory factors, such as IL-1β, which further promote the inflammatory response [24]. Studies have shown that when cells undergo pyroptosis, PI can cross the pores in the cell membrane to stain the nucleus red [25]. Fink et al. reported that *Salmonella* infection of mice caused pyroptosis of host macrophages, with the formation of membrane pores (1.1–2.4 nm) causing cell swelling due to disruption of the osmotic gradient [26]. LDH is an important enzyme in energy metabolism. LDH is released and activity increases as a result of cell death In this study, after infection of macrophages with the pathogenic *E. coli* HPI, the increase of LDH concentrations in the three experimental groups indicated changes in the membrane permeability. The IOD value of PI staining and the expression of GSDMD mRNA were significantly increased, with higher values in the HPI^+^ infection group than that in the HPI^−^. This indicated that *E. coli* HPI infection promoted the formation of pores in the cell membrane of macrophages, and this effect was more marked in the presence of the HPI *irp2* gene. This is consistent with the observation that the number of PI-positive macrophages increased with time after infection with listeria [27].

Muruve and Lamkanfi observed clear signs of pyroptosis and marked increases in the relative mRNA levels of NLRP3, caspase-1, IL-1β and IL-18 after LPS/ATP stimulation of mouse macrophages [28, 29]. Liang reported that the mRNA expression levels of pyroptosis-related genes were increased significantly in renal tissues after obstructive nephropathy caused by unilateral ureteral ligation in rats [30]. In this study, after pathogenic *E. coli* infection of macrophages in vitro, the intracellular mRNA levels of NLRP3, ASC, caspase-1, IL-1β and IL-18 were higher than those in the control group, with an overall increase in levels observed initially followed by decreased expression, which was consistent with previous reports. The findings indicate that *E. coli* activates the pyroptosis signaling pathway, and this effect is promoted more effectively by *E. coli* HPI. In this study, caspase-1 protein expression varied with the different treatments. NLRP3 and caspase-1 protein expression in the LPS + ATP group and the HPI groups increased gradually with time post-infection, which is similar to the pattern of increased caspase-1 protein content in human monocyte macrophages following *Helicobacter pylori* infection [31]. Furthermore, it has been reported that *Neisseria gonorrhoeae* infection promoted the activation and secretion of caspase-1 in human monocytes [32]. Our study confirmed that pathogenic *E. coli* HPI induces pyroptosis in macrophages, and caspase-1 protein expression was higher in host cells infected HPI^+^ strains containing the HPI *irp2* marker gene.

IL-1β and IL-18 play an inflammatory role by binding to the corresponding receptors to regulate the release of soluble antagonists and the expression of precursor enzymes and bait receptors at the transcriptional level. IL-1β and IL-18 are synthesized as inactive cytoplasmic and maturation depends on the caspase-1 activity [33]. The mature forms of IL-1β and IL-18 are released through the GSDMD channel to perform their pro-inflammatory functions and cause pyroptosis [28]. Hitzler showed that *H. pylori* infection of dendritic cells resulted in activation of caspase-1 and induced the maturation and secretion of IL-1β and IL-18 [34].

The results of our study demonstrated that IL-1β/IL-18 levels were significantly elevated in the culture supernatants of macrophages infected with *E.coli* HPI, with higher levels detected following infection with the strain carrying the *irp2* gene. These findings provide evidence of the initial activation of the NLRP3/caspase-1 pyroptosis pathway in cells following HPI infection, which further promoted the secretion of inflammatory factors IL-1β and IL-18. This is consistent with the significant increase in IL-1β and IL-18 expression after LPS/ATP stimulation of mouse macrophages reported by Wei [35]. These findings effectively confirm that HPI endows characteristics of strong pathogenicity on *E. coli*, which is closely related to the process of infection.

## Conclusion

In conclusion, we found that pathogenic *E. coli* HPI infection of Yunnan Saba pigs upregulated the expression of NLRP3, caspase-1, IL-1β and IL-18 mRNA, and promoted cell membrane pore formation and nuclear DNA damage in macrophages. Furthermore, we showed that the infection stimulated the release of inflammatory cytokines IL-1β and IL-18, induce inflammation, and eventually promoted pyroptosis of macrophages. Moreover, the existence of HPI in *E. coli* enhanced the occurrence of pyroptosis of macrophages compared with the effects observed following infection with the HPI^−^ strain. The correlation of *E.coli* HPI infection with the expression of key pyroptosis-related molecules in monocytes highlights new ideas and directions for further studies to elucidate the molecular mechanism by which *E. coli* HPI induces pyroptosis in macrophages. Further studies of pyroptosis will contribute to an improved understanding of the mechanisms of cellular injury and the development of pharmaceutical inhibitors of pyroptosis.

## Methods

### Materials and reagents

The pig macrophage strain 3D4/21 was obtained from BeNa Culture Collection (Beijing). The sampling of *E. coli* strains was conducted from live Yunnan Saba pigs (a pig farm in Chuxiong City, Yunnan Province, China) through fecal swabs. HPI^+^ and HPI^−^ strains of pathogenic *E. coli* were isolated, identified and preserved by the department of animal pathology (Yunnan Agricultural University) [36]. The HPI gene of *E.coli* was identified by polymerase chain reaction (PCR). HPI^+^ and HPI^−^ strains have the same serotype (O119) and biochemical characteristics which were tested by the method reported by Jing et al. [37]. Alcohol and chloroform were from Sichuan Xilong Chemical Industry Group Co., Ltd. (Chengdu, China).

### PCR detection of HPI irp2 gene and culture of macrophages

The *E. coli* HPI strains isolated from Saba pigs were cultured on MacConkey agar medium overnight at 37 °C. Individual colonies of pathogenic *E. coli* were selected, inoculated and cultured on Luria-Bertani (LB) agar plates overnight at 37 °C. At OD_600_ 0.8, bacterial suspensions (containing HPI^+^/ HPI^−^), expression of the HPI *irp2* genes was analyzed by PCR using the *irp2* primers (Table 1). The PCR conditions were as follows: 95 °C 5 min; 94 °C 30 s, 55 °C 30 s, 72 °C 1 min (32 cycles) and one final extension step of 72 °C 8 min. The PCR products were resolved by 1.0% (wt/vol) agarose gel electrophoresis. Macrophages were cultured in DMEM medium containing 10% FBS, 100 U/mL penicillin and streptomycin and incubated at 37 °C under 5% CO_2_.

**Table 1:** Specific primers for amplification of target genes and β-actin gene

Name	Sequences	Primer Length	Tm (°C)	Size (bp)
**β-actin** AY550069.1	5′-TGCGGGACATCAAGGAGA-3′ (F)	18	55	175
	5′-AGGAAGGAGGGCGGAAGAG-3′ (R)	20		
**NLRP3** JQ219660.1	5′-TGGATAGCGGCAAGAGT-3′ (F)	17	47	145
	5′-GCAGCCAGTGAGCAGAG-3′ (R)	17		
**ASC** CV877895.1	5′-GCCATAAACCGGGTTCCTGA-3′ (F)	20	60	148
	5′-CCCATCCACTCTTGGCCATT-3′ (R)	20		
**Caspase-1** NM_214162.1	5′-GCCTTGCCCTCATAATCT-3′ (F)	18	60	282
	5′-ACATCTGGGACTTCTTCG-3′ (R)	18		
**IL-18** AY450287.1	5′-GGATATGCCTGATTCTGACTGTT-3′ (F)	19	50	100
	5′-GATGGTTACTGCCAGACCTCTA-3′ (R)	19		
**IL-1β** NM_214055.1	5′-GCAGTGGAGAAGCCGATGA-3′ (F)	19	62	223
	5′-GGTGGAGAGCCTTCAGCAT-3′ (R)	19		
**GSDMD** AK394823.1	5′-CCCCTTCTACTTCCATGACACT-3′ (F)	22	54	256
	5′-CCTCCGTCACCACGAACAC-3′ (R)	19		

### MTT assay of cell viability

Macrophages in the control and the experimental groups seeded into 96-well plates at 2 × 10^5^/well with five duplicate wells for each group. Medium (100 μL) containing different concentrations of LPS (10^6^, 10^5^, 10^4^, 10^3^, 10^2^ and 10 ng/mL) was added to each well. After 5.5 h, 10 μL ATP (55 mmol/L) was added. After 24 h, 10 μL MTT reagent (5 mg/mL) was added. After a further 4 h, the culture supernatant was removed and 100 μL DMSO was added. Then absorbance at 490 nm was recorded after 15 min of oscillation. The IC_50_ for LPS was calculated using the Improved Kou method as follows:

$$ \lg\;IC50= Xm-\frac{I\left[P-\left(3- Pm- Pn\right)\right]}{4} $$

Xm:lg maximum dose; I:lg (maximum dose/ phase dose); P: sum of positive response rates; Pm: maximum positive response rate; Pn: minimum positive response rate.

### Macrophages infected with *E. coli*

The macrophages were randomly divided into four groups: control, HPI^+^ infection, HPI^−^ infection and LPS + ATP, with three replicates for each group. Macrophages were seeded in 6-well plates at 2 × 10^6^/well, inoculated with HPI^+^ or HPI^−^ strain at a MOI (multiplicity of infection) of 1 for the indicated time, and LPS + ATP (2 mL LPS at 4.79 ng/mL, 100 μL ATP at 100 mmol/L). At 0.5, 3, 6 and 9 h post-infection, macrophages and their supernatant were collected for analysis.

### PI staining and lactate dehydrogenase (LDH) assay

PI staining: After pretreatment, macrophages were seeded in 6-well plates (2 × 10^6^/well) and fixed with 4% paraformaldehyde on ice for 15 min. The cells were cultured with 100 μL of 6.7 μg/mL PI staining solution at 37 °C for 20 min. Macrophages were observed under a light microscope at 20 × objective (Olympus IX73P1F microscope, Japan).

LDH assay: Macrophages were prepared, then the supernatants from cells in each group were collected, the contents of the LDH was detected according to the instructions provided on the kits (Nanjing Jiancheng Bioengineering Institute, Nanjing, China).

### Quantitative real time PCR (q-PCR)

After pretreatment of macrophages, total RNA was extracted, cDNA was synthesized using q-PCR reverse transcription Kits (TaKaRa, Dalian, China) and stored at − 20 °C. The q-PCR reaction was carried out using gene-specific primers for β-actin, NLRP3, caspase-1, IL-1β and IL-18 (Table 1) with SYBR Premix Ex TaqTM II (TaKaRa, Dalian, China) according to the manufacturer’s instructions. The amplification and detection procedures were carried out using the Bio-Rad Cx96 Detection System (Shanghai, China). The relative mRNA expression level of each index gene was calculated using the 2^-△△Ct^ method.

### Immunofluorescence assay (IFA) of HPI on NLRP3 and caspase-1 protein expression into macrophages

After pretreatment, macrophages were seeded in 6-well plates (2× 10^6^/well), fixed, permeabilized and then incubated overnight at 4 °C with anti-NLRP3 (rabbit ployclonal, 1:200, Absin Bioscience Inc., Shanghai, China) and anti-caspase-1 (mouse monoclonal, 1:50, Santa Cruz Biotechnology USA), respectively. Then cells were incubated in the dark with secondary detection antibodies before 4′, 6-diamidino-2-phenylindole (DAPI) treatment. The IOD values for NLRP3 and caspase-1 protein expression were analyzed using Image Pro-Plus 6.0 software (Media Cybernetics, Silver Spring, MD, USA). Positive cell rate (%) = Average number of positive cells /Number of DAPI cells × 100.

### Elisa

Macrophages were prepared as described in section lactate dehydrogenase (LDH) assay. The supernatants from cells in each group were collected and the contents of the inflammatory mediators IL-1β/IL-18 were detected by commercially available Porcine IL-1β/IL-18 ELISA kits (Shanghai Yuanye Bio-Technology, Co. Ltd., Shanghai, China) according to the manufacturers’ instructions. The coefficient variability of intra-assay and inter-assay was less than 10%.

### Statistical analysis

All experimental data were presented as means ± SD (*n* = 3) with one-way ANOVA, followed by the Duncan post-hoc test used to analyze differences between groups (SPSS, version 19.0, SPSS Science, Chicago, USA). Differences were regarded as significant at *P* < 0.05, or extremely significant *P* < 0.01.

## Data Availability

The datasets used and/or analysed during the current study are available from the corresponding author on reasonable request.

## Abbreviations

*E. coli*: *Escherichia coli*
HPI: High pathogenicity island
q-PCR: Quantitative real time PCR
Ybt: Yersiniabactin
PCD: Programmed cell death
GSDMD: Gasdermin D
PI: Propidium iodide
IFA: Immunofluorence assay
LDH: Lactate dehydrogenase
NLRP3: NOD-like receptor family pyrin domain-containing 3
ASC: Apoptosis-associated speck-like protein
CARD: Caspase activation and recruitment domains
pro-IL-1β: Pro-interleukin-1β
pro-IL-18: Pro-interleukin-18
